# The m6A-regulation and single cell effect pattern in sunitinib resistance on clear cell renal cell carcinoma: Identification and validation of targets

**DOI:** 10.3389/fphar.2023.1131610

**Published:** 2023-03-31

**Authors:** Yanxi Deng, Fang Wang, Xinhui Wu, Kangming Du, Qing Yang, Ting Xia

**Affiliations:** ^1^ Clinical Laboratory, Hospital of Chengdu University of Traditional Chinese Medicine, Chengdu, Sichuan, China; ^2^ Hospital of Chengdu University of Traditional Chinese Medicine, Chengdu, Sichuan, China; ^3^ Department of Cardiothoracic Surgery, Hospital of Chengdu University of Traditional Chinese Medicine, Chengdu, Sichuan, China; ^4^ Chengdu University of Traditional Chinese Medicine, Chengdu, Sichuan, China

**Keywords:** epigenetic, sunitinib, ccRCC, MX2, biological

## Abstract

**Background:** Sunitinib is the main target drug for clear cell renal cell carcinoma. However, the effect of sunitinib is often limited by acquired drug resistance.

**Methods:** The open-accessed data used in this study were obtained from different online public databases, which were analyzed using the R software. The RNA level of specific genes was detected using quantitative Real-Time PCR. Sunitinib-resistant cell lines were constructed based on protocol get from the previous study. Colony formation and Cell Counting Kit-8 assays were applied to detect cell proliferation ability.

**Results:** In this study, through publicly available data and high-quality analysis, we deeply explored the potential biological mechanisms that affect the resistance of sunitinib. Detailed, data from GSE64052, GSE76068 and The Cancer Genome Atlas were extracted. We identified the IFITM1, IL6, MX2, PCOLCE2, RSAD2 and SLC2A3 were associated with sunitinib resistance. Single-cell analysis, prognosis analysis and m6A regulatory network were conducted to investigate their role. Moreover, the MX2 was selected for further analysis, including its biological role and effect on the ccRCC microenvironment. Interestingly, we noticed that MX2 might be an immune-related gene that could affect the response rate of immunotherapy. Then, *in vitro* experiments validated the overexpression of MX2 in sunitinib-resistance cells. Colony formation assay indicated that the knockdown of MX2 could remarkably inhibit the proliferation ability of 786-O-Res and Caki-1-Res when exposed to sunitinib.

**Conclusion:** In summary, through publicly available data and high-quality analysis, we deeply explored the potential biological mechanisms that affect the resistance of sunitinib. MX2 was selected for further analysis, including its biological role and effect on the ccRCC microenvironment. Finally, *in vitro* experiments were used to validate its role in ccRCC.

## Introduction

Renal cell carcinoma (RCC) is a malignant tumor that arises out of the renal tubular epithelium, which is very common in the world. It is estimated that about 300,000 new cases are created each year, and 130,000 cancer-related deaths are caused at the same time (Cohen and McGovern, 2005). Among them, seventy to eighty percent of all cases of renal cell carcinoma are clear cell (ccRCC) (Wettersten et al., 2017). As a multifactorial disease, the pathogenesis of ccRCC is not completely clear, and it is closely related to age, obesity, smoking, hypertension, genetic factors, and so on (Znaor et al., 2015). In the past decade, the incidence rate of renal tumors worldwide has shown a trend of continuous growth, and the internal microenvironment of tumors is usually accompanied by the reprogramming of metabolic networks and pathways. Through metabolic reprogramming, tumor cells proliferate rapidly, survive under hypoxia and nutrient depletion, and escape immune surveillance (Wettersten et al., 2017). Meanwhile, due to the lack of early clinical symptoms, more than 30%–50% of ccRCC patients missed the best opportunity for surgery, making the diagnosis, treatment, and prevention of it a serious public health problem worldwide.

At present, for early and resectable RCC, radical surgery is still a major treatment. Although surgery can cure most early-stage patients, due to the high blood metastasis rate, about 25% of locally progressed or localized patients will suffer from metastasis (Dudani et al., 2021). Additionally, considering the occult symptoms of RCC, about 20%–25% of patients had distant metastasis at the time of diagnosis and could not undergo radical surgery (Xue et al., 2021). Moreover, for patients with advanced or metastatic stages, the five-year survival rate is only about 23% due to the high heterogeneity and invasiveness of the disease (Atkins and Tannir, 2018). Unfortunately, the treatment of ccRCC with radiotherapy and chemotherapy is not effective, which limits its treatment options to some extent. Sunitinib is a kind of drug that can selectively target tyrosine kinase, which is widely utilized in RCC and has achieved encouraging results (Bex et al., 2019). However, some patients receiving sunitinib treatment will still be limited by acquired drug resistance (McDermott et al., 2018). Considering the practical significance of this problem, researchers have begun to pay attention to and identify the specific biological mechanism of acquired resistance to sunitinib (Broxterman et al., 2009). Zhu and their colleagues found that the ZHX2 can induce sunitinib resistance through the autophagy regulated by MEK/ERK axis (Zhu et al., 2020). Bender and their colleagues noticed that the overexpressed PRKX, TTBK2 and RSK4 can lead to sunitinib resistance (Bender and Ullrich, 2012). The m6A RNA methylation is an epigenetic modification pathway widely existing in the cancer microenvironment. Chen and their colleagues revealed that TRAF1 can contribute to sunitinib resistance based on the METTL14 and m6A modifications (Chen et al., 2022). Consequently, it is of practical clinical significance to identify biological targets that may participate in the resistance of sunitinib.

Access to public data can provide convenience for researchers (Wang et al., 2019a; Wei et al., 2020; Zhang et al., 2022). Here, through publicly available data and high-quality analysis, we deeply explored the potential biological mechanisms that affect the resistance of sunitinib. Detailed, data from GSE64052, GSE76068 and TCGA were extracted. We identified the IFITM1, IL6, MX2, PCOLCE2, RSAD2 and SLC2A3 were associated with sunitinib resistance. Single-cell analysis, prognosis analysis and m6A regulatory network were conducted to investigate their role. Moreover, the MX2 was selected for further analysis, including its biological role and effect on the ccRCC microenvironment. Interestingly, we noticed that MX2 might be an immune-related gene that could affect the response rate of immunotherapy. Then, *in vitro* experiments validated the overexpression of MX2 in sunitinib-resistance cells. Colony formation assay indicated that the knockdown of MX2 could remarkably inhibit the proliferation ability of 786-O-Res and Caki-1-Res when exposed to sunitinib.

## Methods

### Acquisition and pre-processing of open-accessed data

The open-accessed data used in this study were obtained from the Gene Expression Omnibus (GEO) and The Cancer Genome Atlas (TCGA) databases. The GSE64052 and GSE76068 contain the sequence information between the sunitinib-resistant and wild-type RCC cells (Zhang et al., 2015). For the TCGA database, the clinical features and transcription profile information were directly downloaded from the TCGA-KIRC project. Initially, the expression profile file of the individual patient was downloaded in “STAR-Counts” format and converted into TPM format through R code. Before analysis, we adjusted the range of expression values to 1–20 through data preprocessing for all data. The first step is to annotate the probe ID as the corresponding gene symbol through the annotation file (GRCh38. p13). The second part is to complete the missing values in the expression matrix. The third step is to average the expression amount of duplicate gene symbols and remove the part where the mean value is less than 0.1. Limma package was applied to identify the genes differentially expressed between different groups (Ritchie et al., 2015). The genes affected by sunitinib were get from the CTD database. The baseline information of TCGA-KIRC patients was shown in Supplementary Table S1.

### Gene ontology (GO) and kyoto encyclopedia of genes and genomes (KEGG)

GO and KEGG analysis can reflect the biological effect based on the input molecules, which was performed using the clusterprofiler package (Yu et al., 2012). Detailed, the “OrgDb” was “org.Hs.eg.db”; the “pvalueCutoff” was 0.05; the “qvalueCutoff” was 0.05; the “ont” was “all”.

### Single-cell evaluation

Specific gene expression patterns in the ccRCC microenvironment were evaluated using the online website TISCH project, a scRNA-seq database aiming to characterize tumor microenvironment at single-cell resolution (Sun et al., 2021). Detailed, the database in TISCH projects KIRC_GSE111360, KIRC_GSE121636, KIRC_GSE139555 and KIRC_GSE145281 were selected to illustrate the single-cell expression pattern of MX2 (major-linegae).

### Cytoscape software

The co-expression analysis was visualized using the Cytoscape software (Shannon et al., 2003).

### Pathway investigation

To identify pathways significantly different between the two groups, gene set enrichment analysis (GSEA) was employed. Reference gene set was “Hallmark”. The enriched pathways with false discovery rate (FDR) < 0.25 and p.adjust < 0.05 were regarded as significant (Subramanian et al., 2005). Based on the pathway set, single sample GSEA (ssGSEA) analysis was conducted (Hänzelmann et al., 2013).

### Methylation

The list of molecules involved m6A process was collected from the previous study (Lv et al., 2021). The correlation between clinical features and gene methylation was investigated using the MEXPRESS database (https://mexpress.be/).

### Tumor microenvironment

Through bioinformatics analysis, the tumor microenvironment can be quantified using specific algorithms. In our study, the tumor microenvironment was quantified using the EPIC, MCPCOUNTER, TIMER, CIBERSORT, QUANTISEQ and XCELL algorithms (Becht et al., 2016; Li et al., 2017; Chen et al., 2018; Racle and Gfeller, 2020).

### Tumor immune dysfunction and exclusion (TIDE)

The TIDE score quantified by the TIDE algorithm can reflect the response rate of patients on immunotherapy. Meanwhile, as well as immune dysfunction and immune exclusion levels, the TIDE algorithm quantified cancer-associated fibroblasts, M2 macrophages, and myeloid-derived suppressor cells (Fu et al., 2020).

### Immunohistochemistry

In the HPA database, MX2 was immunohistochemically detected in ccRCC tumors and normal tissue (Uhlén et al., 2015).

### Establishment of sunitinib-resistant cell lines and cell culture

The 786-O and Caki-1 cell lines were laboratories stored and cultured in RPMI-1640 culture medium added with 10% fetal bovine serum (FBS) under the standard cell culture conditions of 37°C with 5% CO_2_. The process to induce the cell lines resistant to sunitinib was followed by a previous study (Sakai et al., 2013; Wei et al., 2021). The IC50 of used cells 786-O/786-O-Res and Caki-1/Caki-1-Res were 27.66/102.1 and 10.26/73.59 nM.

### Quantitative Real-Time PCR

Total RNA extraction and cDNA preparation were conducted following the standard process (Wei et al., 2021). The primer used for PCR was: forward, 5′-TGA​ACG​TGC​AGC​GAG​CTT-3′, reverse, 5′-GGCTT GTGGGCCTTAGACAT-3′; GPADH, 5′-CTG​GGC​TAC​ACT​GAG​CAC​C-3’; reverse, 5′-AAG​TGG​TCG​TTG​AGG​GCA​ATG-3’.

### RNA interference

The plasmids used for cell transfection were purchased from Shanghai GenePharma Co., Ltd., and the sequences were: sh#1: 5′-GCA​CGA​TTG​AAG​ACA​TAA​A-3′, sh#2: 5′- GGG​ACG​CCT​TCA​CAG​AAT​A-3′, sh#3: 5′-GCC​AAC​CAG​ATC​CCA​TTT​A-3’. The processes of cell transfection were conducted following the standard process using the Lipofectamine 3,000 regrant.

### Cell Counting Kit-8 (CCK8) and colony formation assays

CCK8 and colony formation assays were conducted following the standard process (Wei et al., 2021).

### Statistical analysis

All the analysis were completed in the R, SPSS and GraphPad Prism 8 software. The 0.05 was set as the statistical threshold. Normally distributed data are analyzed using independent T-tests. Non-normally distributed data are analyzed using the Mann-Whitney U tests.

## Results

**FIGURE 1:**
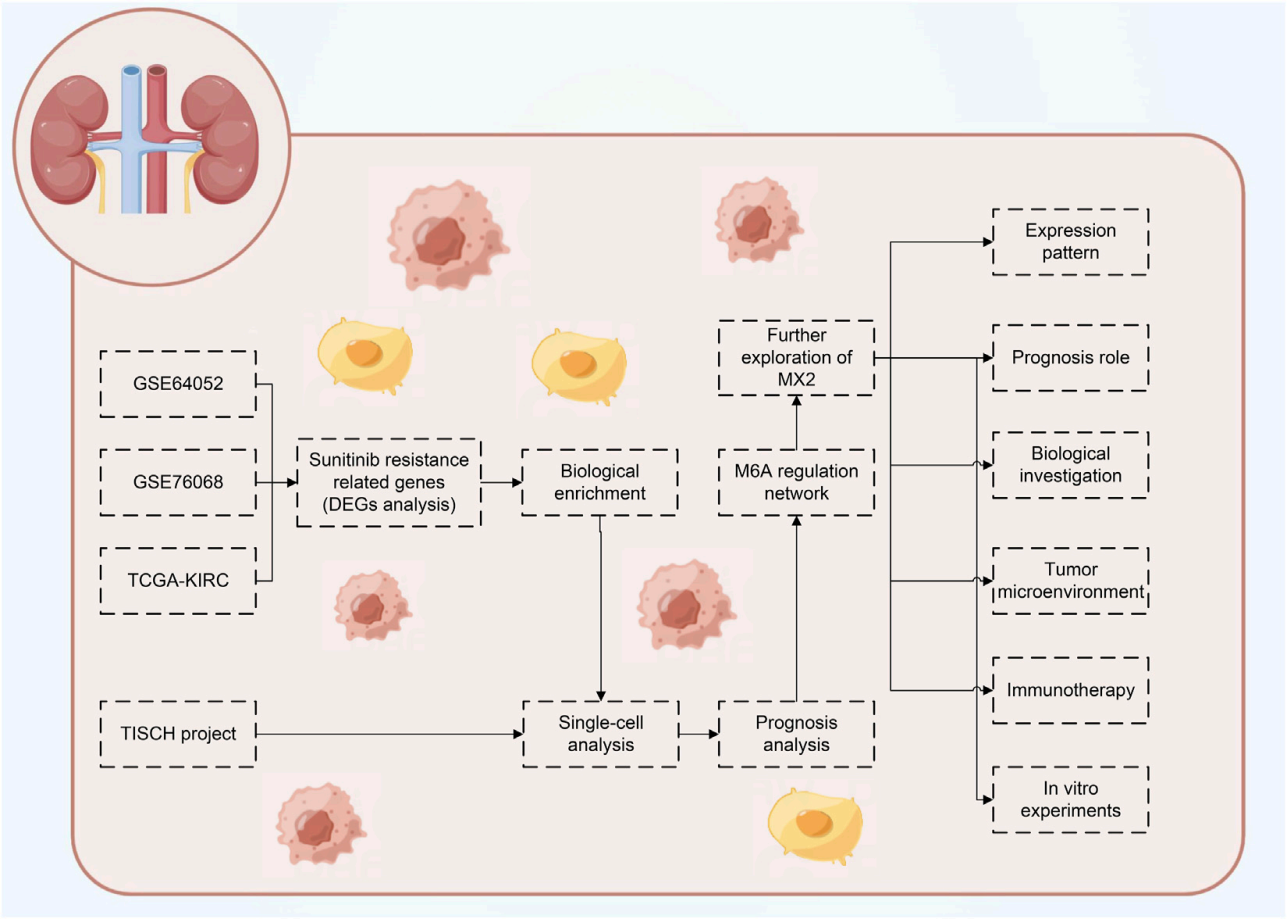
The flow chart of the whole study.

Figure 1 illustrates the flow chart of our study. In this study, we identified the molecules involved in sunitinib resistance through the data from GSE64052, GSE76068 and TCGA-KIRC. Then, the biological enrichment and single-cell analysis based on TISCH project were conducted to investigate the role of identified molecules in ccRCC, as well as their interaction network with m6A regulators. Ultimately, MX2 was identified for further analysis, including expression pattern, prognosis role, biological investigation, tumor microenvironment, immunotherapy evaluation and *in vitro* experiments validation.

### Effect of sunitinib-resistant related genes in ccRCC

**FIGURE 2:**
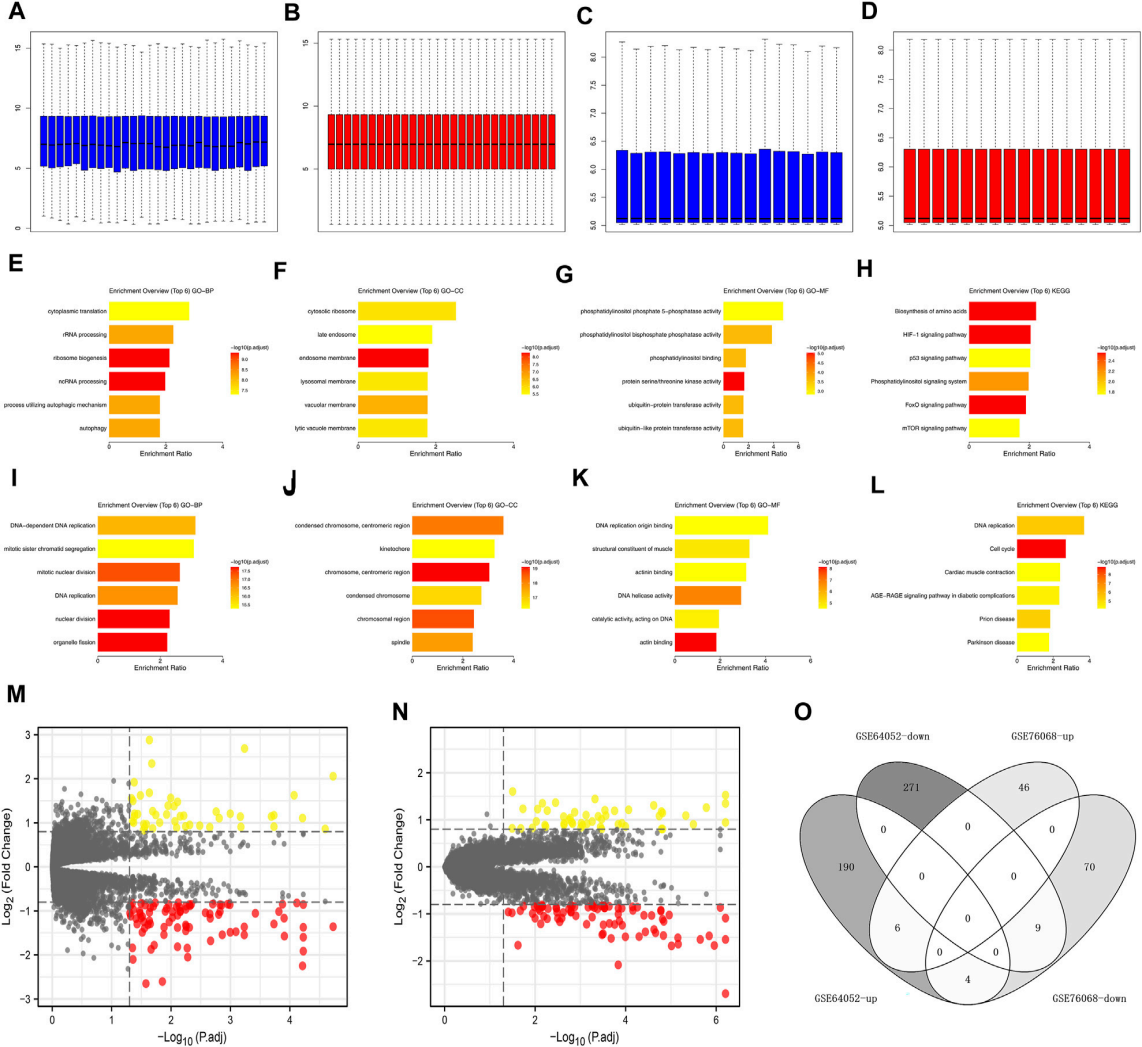
Effect of sunitinib-resistant related genes in ccRCC. **(A)** The GO-BP terms of genes positively correlated with sunitinib (CTD database); **(B)** The GO-CC terms of genes positively correlated with sunitinib (CTD database); **(C)** The GO-CC terms of genes positively correlated with sunitinib (CTD database); **(D)** The KEGG terms of genes positively correlated with sunitinib (CTD database); **(E)** The GO-BP terms of genes negatively correlated with sunitinib (CTD database); **(F)** The GO-CC terms of genes negatively correlated with sunitinib (CTD database); **(G)** The GO-CC terms of genes negatively correlated with sunitinib (CTD database); **(H)** The KEGG terms of genes negatively correlated with sunitinib (CTD database); **(I,J)** Data normalization of GSE64052; **(K,L)** Data normalization of GSE76068; **(M)** DEGs analysis of GSE64052; **(N)** DEGs analysis of GSE76068; **(O)** Intersection of DEGs result of GSE76068 and GSE64052.

The data normalization process of GSE64052 and GSE76068 were shown in Figures 2A–D. We investigated the underlying biological effects of sunitinib on ccRCC cells. For genes positively correlated with sunitinib (Supplementary Material S1), the genes were enriched in cytoplasmic translation, rRNA processing, ribosome biogenesis, ncRNA processing, process utilizing autophaic mechanism and autophagy (Figure 2E, GO-BP); cytosolic ribosome, late endosome, lysosomal membrane, vacuolar membrane and lytic vacuole membrane (Figure 2F, GO-CC); phosphatidylinositol binding, ubiquitin-protein transferase activity and ubiquitin-like protein transferase activity (Figure 2G, GO-MF); biosynthesis of amino acids, HIF-1 signaling pathway, p53 signaling pathway, phosphatidylinositol signaling system, FoxO signaling pathway and mTOR signaling pathway (Figure 2H, KEGG). For genes negatively correlated with sunitinib (Supplementary Material S1), the genes were enriched in DNA-dependent DNA replication, mitotic sister chromatid segregation, mitotic nuclear division, DNA replication, nuclear division, and organelle fission (Figure 2I, GO-BP); centromeric region, condensed chromosome, chromosomal region and spindle (Figure 2J, GO-CC); DNA replication origin binding, structural constituent of muscle, actinin binding, DNA helicase activity, catalytic activity, acting on DNA and actin binding (Figure 2K, GO-MF); DNA replication, cell cycle, cardiac muscle contraction, age-race signaling pathway in diabetic complications, prion disease and parkinsion disease (Figure 2L, KEGG). Through the limma package with the threshold of |log FC| > 0.5 and *p* < 0.05, 280 downregulated and 200 upregulated genes were identified in GSE64052 between the sunitinib-resistant and wild-type RCC cells (Figure 2M); 83 downregulated and 53 upregulated genes were identified in GSE76068 between the sunitinib-resistant and wild-type RCC cells (Figure 2N). Furthermore, we found that six genes were commonly upregulated, while nine genes were commonly downregulated in both GSE64052 and GSE76068 cell lines (Figure 2O). The clinical roles of these six genes were shown in Supplementary Figure S1A–D.

### Single-cell analysis

**FIGURE 3:**
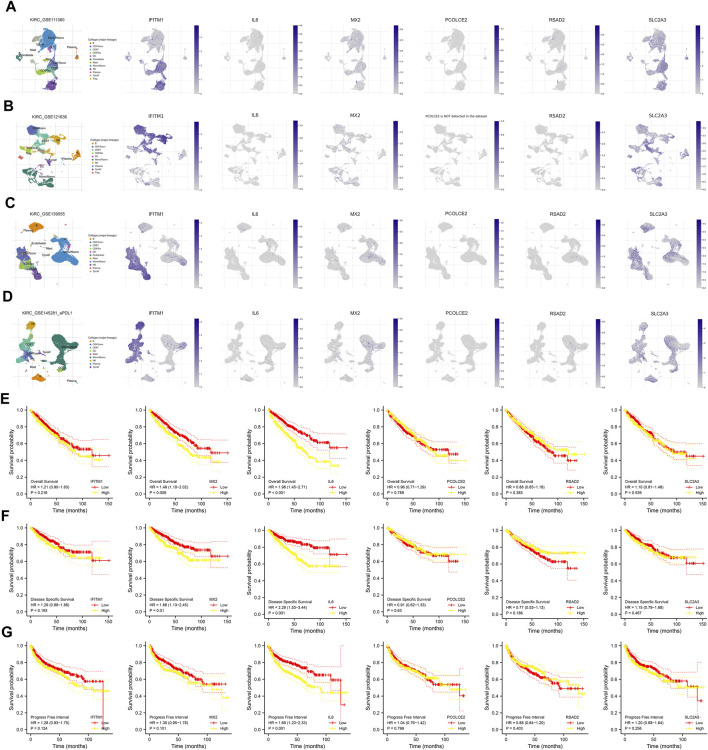
Single-cell and prognosis analysis of IFITM1, IL6, MX2, PCOLCE2, RSAD2 and SLC2A3. **(A)** Single-cell analysis of IFITM1, IL6, MX2, PCOLCE2, RSAD2 and SLC2A3 in GSE111360; **(B)** Single-cell analysis of IFITM1, IL6, MX2, PCOLCE2, RSAD2 and SLC2A3 in GSE121636; **(C)** Single-cell analysis of IFITM1, IL6, MX2, PCOLCE2, RSAD2 and SLC2A3 in GSE139555; **(D)** Single-cell analysis of IFITM1, IL6, MX2, PCOLCE2, RSAD2 and SLC2A3 in GSE145281; **(E)** Overall survival of IFITM1, IL6, MX2, PCOLCE2, RSAD2 and SLC2A3 in ccRCC; **(F)** Disease free survival of IFITM1, IL6, MX2, PCOLCE2, RSAD2 and SLC2A3 in ccRCC; **(G)** Progression free survival of IFITM1, IL6, MX2, PCOLCE2, RSAD2 and SLC2A3 in ccRCC.

Following this, we evaluated the single-cell level of six commonly upregulated genes in the ccRCC single-cell level (Figures 3A–D; Supplementary Figure S2A–E). Results indicated that IFITM1 was mainly expressed in NK cells, Treg and CD8^+^ T cells in four ccRCC single-cell cohorts, GSE111360, GSE121636, GSE139555 and GSE145281; the overall expression level of IL6, PCOLEC2 and RSAD2 seems to be very low; MX2 and SLC2A3 are expressed in various cells. KM survival curves were then used to identify the prognosis role of these genes (Figures 3E–G). Results indicated that the MX2 and IL6 are associated with worse survival performance of patients, but the statistical *p*-value of IFITM1, PCOLCE2, RSAD2, and SLC2A3 were not significant.

### The m6A-regulation regulatory network of sunitinib-resistant related genes

**FIGURE 4:**
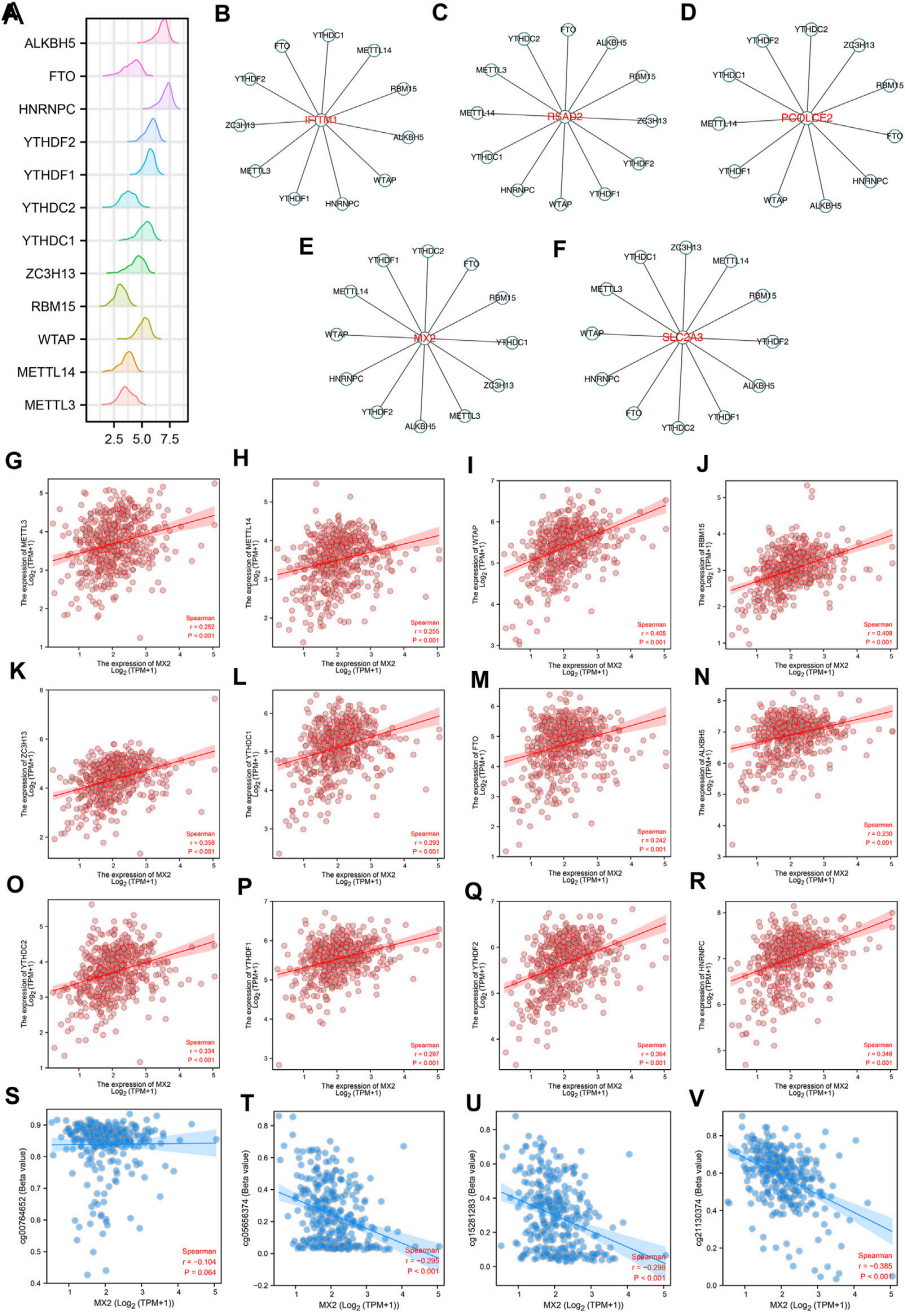
The m6A regulatory network of IFITM1, IL6, MX2, PCOLCE2, RSAD2 and SLC2A3. **(A)** The expression pattern of m6A regulators in ccRCC; **(B–F)** The m6A regulatory network of IFITM1, IL6, MX2, PCOLCE2, RSAD2 and SLC2A3; **(G–R)** Correlation between MX2 and m6A regulators; **(S–V)** Correlation between MX2 and methylation site.

The m6A modification is an important part of the epigenetic field and has been reported to affect sunitinib resistance (Li et al., 2022). The expression pattern of m6A regulators was shown in Figure 4A. We noticed that the IFITM1 was regulated by YTHDC1, METTL14, RBM15, ALKBH5, WTAP, HNRNPC, YTHDF1, METTL3, ZC3H13, YTHDF2 and FTO (Figure 4B); RSAD2 was regulated by YTHDC2, FTO, ALKBH5, RBM15, ZC3H13, YTHDF2, YTHDF1, WTAP, HNRNPC, YTHDC1, METTL14, METTL3 and YTHDC2 (Figure 4C); PCOLCE2 was regulated by YTHDC2, ZC3H13, RBM15, FTO, HNRNPC, ALKBH5, WTAP, YTHDF1, METTL14, YTHDC1, YTHDF2 (Figure 4D); MX2 was regulated by YTHDC2, FTO, RBM15, YTHDC1, ZC3H13, METTL3, ALKBH5, YTHDF2, HNRNPC, WTAP, METTL14 and YTHDF1 (Figure 4E); SLC2A3 was regulated by ZC3H13, METTL14, RBM15, YTHDF2, ALKBH5, YTHDF1, YTHDC2, FTO, HNRNPC, WTAP, METTL3 and YTHDC1 (Figure 4F). Interestingly, we noticed MX2 was positively correlated with all m6A regulators, including HNRNPC, YTHDF2, METTL3, YTHDF1, YTHDC2, ALKBH5, FTO, YTHDC1, ZC3H13, RBM15, WTAP and METTL14 (Figures 4G–R). Moreover, we noticed that the methylation sites cg00764652, cg05656374, cg152811283, and cg21130374 were negatively correlated with the MX2 expression (Figures 4S–V).

### Subsequent analysis of MX2

**FIGURE 5:**
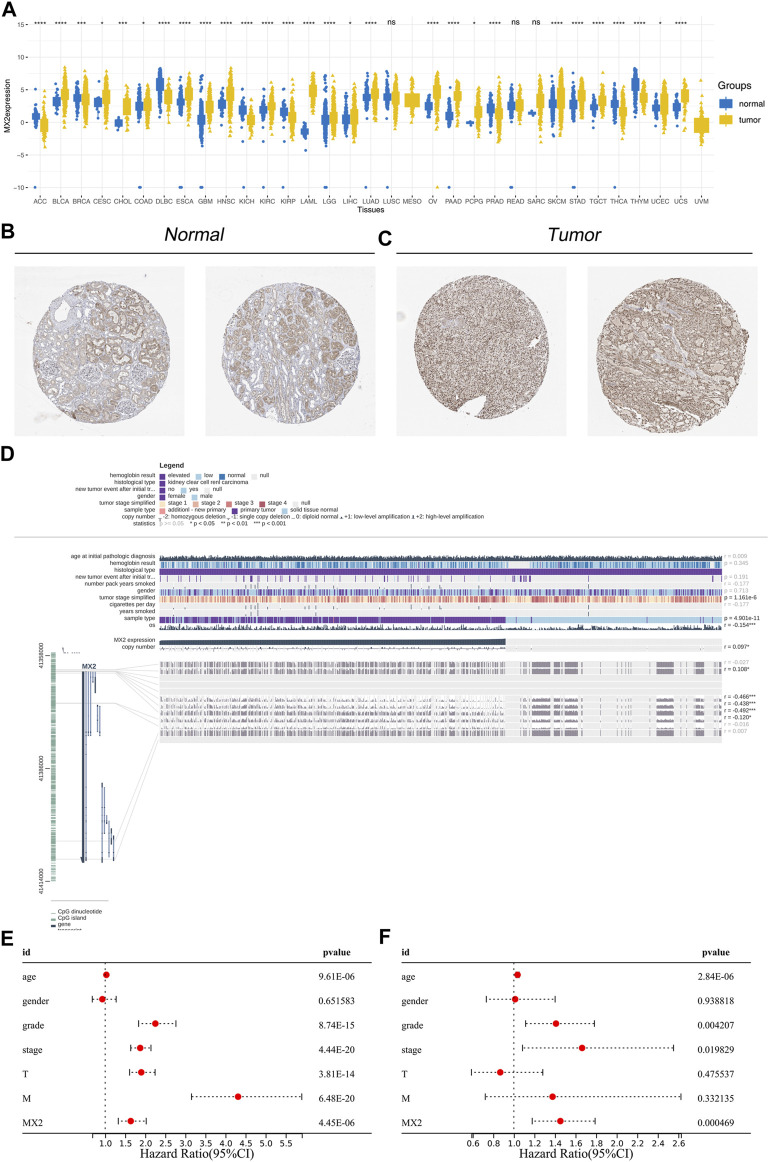
Effect pattern of MX2 in ccRCC. **(A)** Pan-cancer analysis of MX2; **(B)** The immunohistochemistry image of MX2 in normal renal tissue, ns = *p* > 0.05, * = *p* < 0.05, ** = *p* < 0.01, *** = *p* < 0.001, **** = *p* < 0.0001; **(C)** The immunohistochemistry image of MX2 in renal cancer tissue; **(D)** Overview of MX2 methylation in ccRCC, * = *p* < 0.05, *** = *p* < 0.001; **(E)** Univariate Cox regression analysis of MX2; **(F)** Multivariate Cox regression analysis of MX2.

Next, we evaluated the expression pattern of MX2 in pan-cancer. Results showed that MX2 was differentially expressed in most cancer (Figure 5A). We noticed a relatively higher protein level of MX2 in ccRCC protein (Figures 5B, C). The overview of the MX2 and methylation site were shown in Figure 5D. Cox regression analysis of single factor and multiple factors showed that MX2 is an independent prognosis factor for ccRCC survival (Figures 5E, F). We also explored the lncRNAs and mRNAs significantly correlated with MX2 expression, which was shown in Supplementary Material S2, S3.

### Biological investigation

**FIGURE 6:**
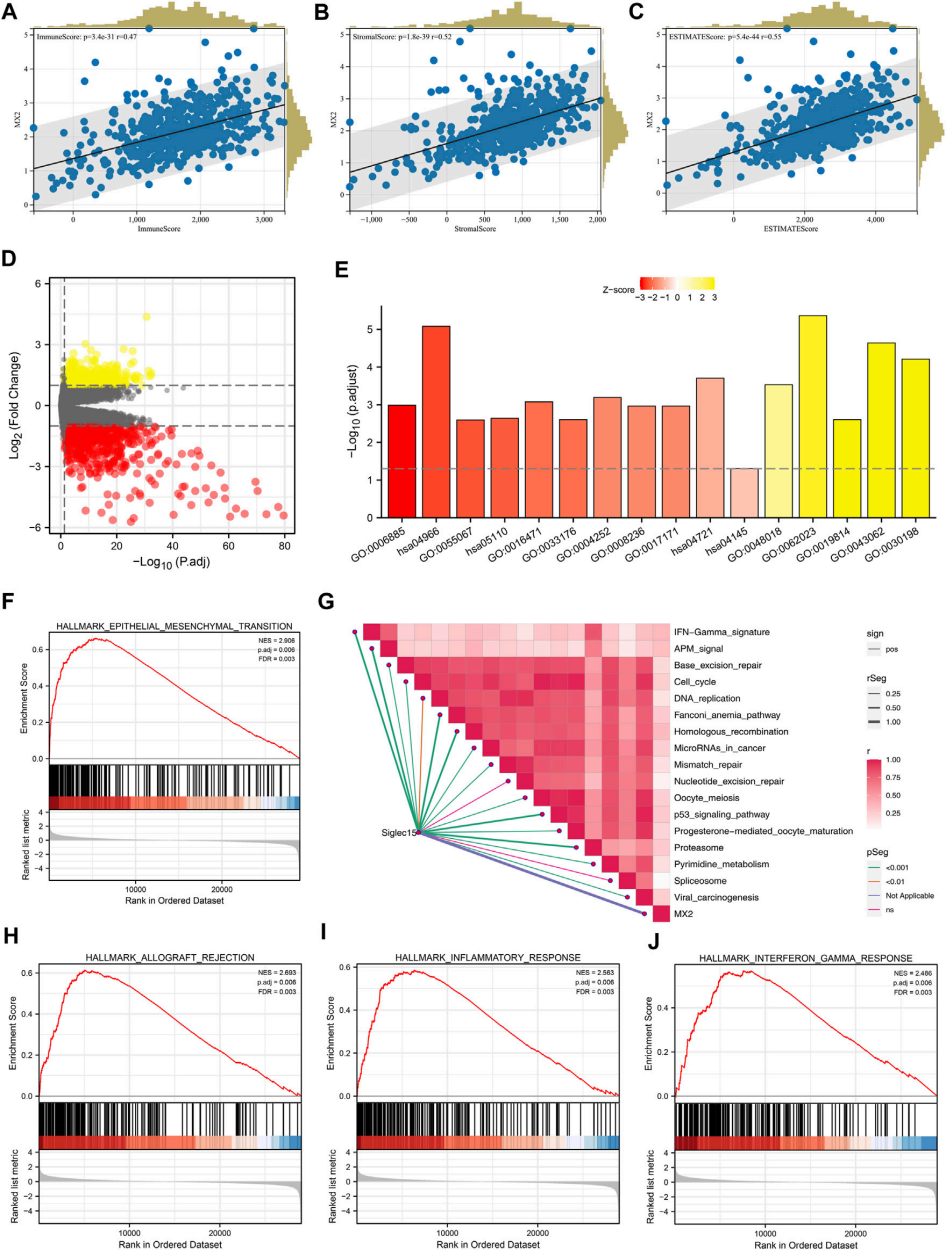
Biological investigation of MX2. **(A–C)** Correlation between MX2 and immune score, stromal score and estimate score quantified by estimate package; **(D)** DEGs analysis in patients with high and low MX2 expression; **(E)** GO and KEGG analysis of MX2 in ccRCC; **(F, H–J)** GSEA analysis based on Hallmark gene set; **(G)** ssGSEA algorithm was used to quantify the enrichment score of immune pathways.

The Estimate R package was utilized to quantify the tumor microenvironment of the ccRCC microenvironment. In the correlation analysis, the immune score, stromal score, and estimate score were positively correlated with the MX2 (Figures 6A–C). The differentially expressed genes (DEGs) analysis was performed between the patients with high and low MX2 expression (Figure 6D). Based on these DEGs, we found MX2 was mainly enriched in the terms of GO:0006885, hsa04966, GO:0055067, hsa05110, GO:0033176, GO:0004252, GO:0008236, GO:0017171, hsa04145, GO:0048018, GO:0019814, GO:0043062, GO:0030198 (Figure 6E). There was a positive correlation between MX2 and multiple pathways in the ssGSEA analysis (Figure 6G). Using GSEA analysis, it was revealed that the DEGs with a high level of epithelial-mesenchymal transition, allograft rejection and inflammation were enriched in the Hallmark signaling (Figures 6F, H–J).

### Effect of MX2 on tumor microenvironment

**FIGURE 7:**
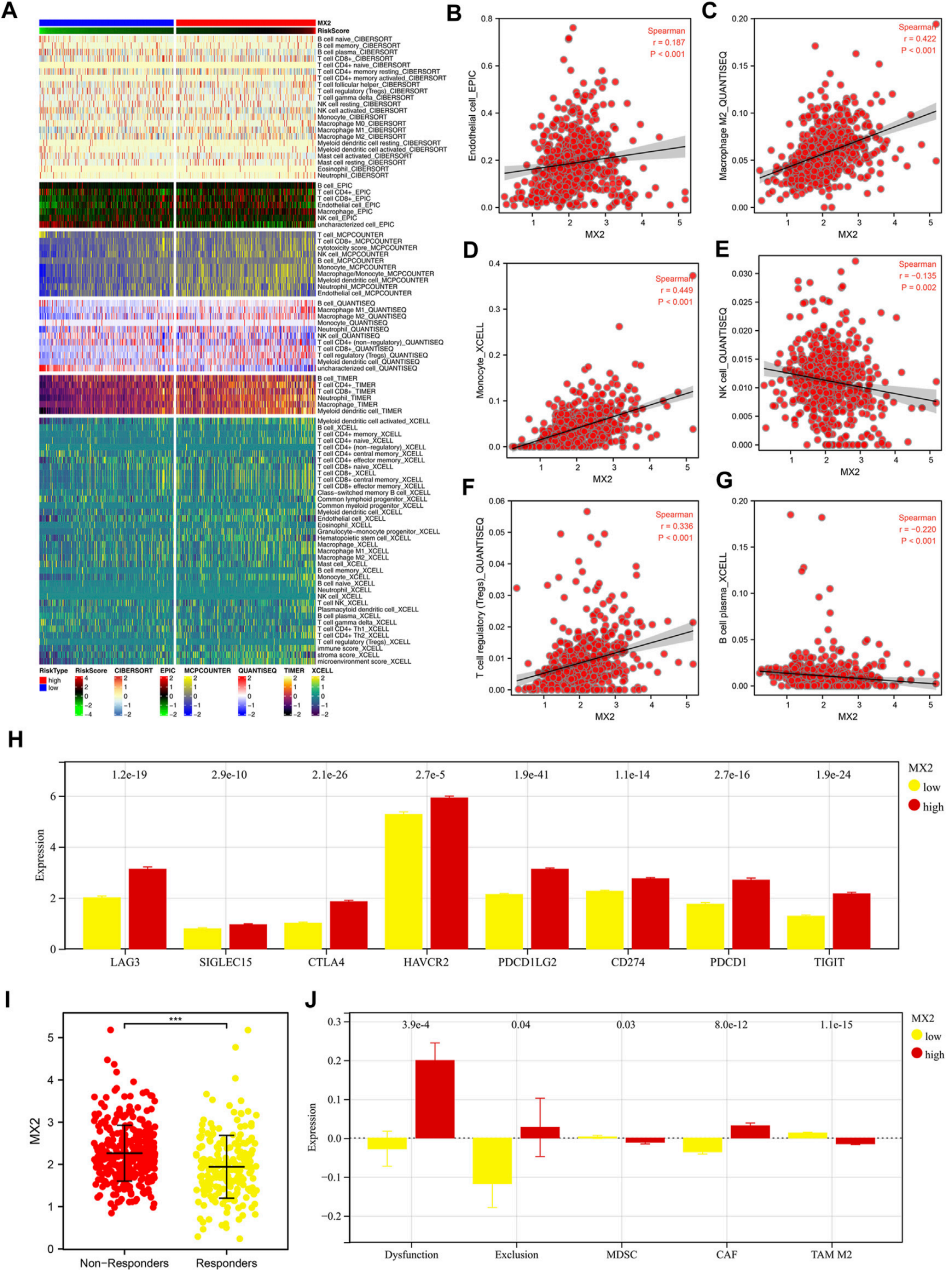
Effect of MX2 on ccRCC microenvironment. **(A)** The ccRCC microenvironment was quantified based on multiple algorithms; **(B–G)** Correlation between MX2 and specific cells; **(H)** The expression level of specific immune checkpoints in patients with high and low MX2 expression; **(I)** The expression level of MX2 in immunotherapy responders and non-responders, *** = *p* < 0.001; **(J)** Levels of immune dysfunction, immune exclusion and CAF, MDSC and TAM M2 in patients with high and low MX2 expression.

Multiple algorithms mentioned in the method section were utilized to quantify the tumor microenvironment of ccRCC. From the heatmap, we observed a remarkably different infiltration pattern of quantified cells in patients with high and low MX2 expression (Figure 7A). Correlation analysis showed that MX2 was positively correlated with endothelial cell_EPIC, macrophages M2_QUANTISEQ, monocyte_XCELL, Tregs_QUANTISEQ, yet negatively correlated with the NK cell_QUANTISEQ and B cell plasma_XCELL (Figures 7B–G). Moreover, we noticed that all the key immune checkpoints, including LAG3, SIGLEC15, CTLA4, HAVCR2, PDCD1LG2, CD274, PDCD1 and TIGIT were overexpressed in patients with high MX2 level (Figure 7H). Furthermore, we tried to explore whether MX2 has an impact on the immunotherapeutic response of ccRCC. Results showed that the immunotherapy non-responders had a higher MX2 level (Figure 7I). Meanwhile, patients with higher MX2 expression might have a higher level of immune dysfunction, immune exclusion and CAF, while a lower level of MDSC and TAM M2 (Figure 7J).

### MX2 is associated with sunitinib resistance

**FIGURE 8:**
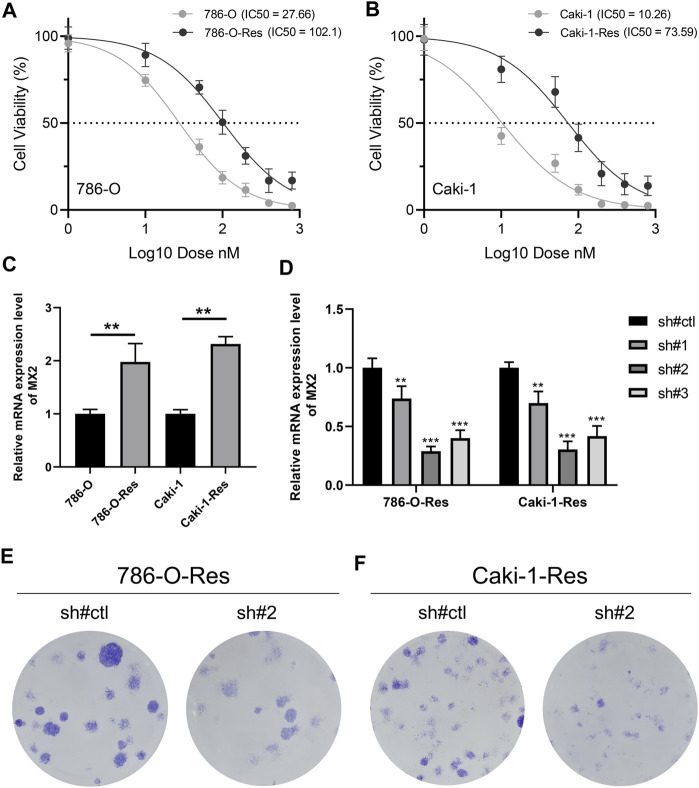
MX2 is associated with sunitinib resistance **(A–B)** The IC50 of wild-type and sunitinib-resistant cells (786-O and Caki-1); **(C)** The expression level of MX2 in wild-type and sunitinib-resistant cells, ** = *p* < 0.01; **(D)** PCR was used to validate the knockdown efficiency of MX2, ** = *p* < 0.01, *** = *p* < 0.001; **(E)** Colony formation assay in 786-O-Res and Caki-1-Res when exposed to sunitinib.

Through the method mentioned above, we construct two cell lines resistant to sunitinib, named 786-O-Res and Caki-1-Res. The results of IC50 to sunitinib validated the resistance of these cells on sunitinib (Figure 8A, IC50 of 786-O-wild = 27.66, IC50 of 786-O-Res = 102.1; Figure 8B, IC50 of Caki-1-wild = 10.26, IC50 of Caki-1-Res = 73.59). The result of the PCR revealed that MX2 was overexpressed in sunitinib-resistance cell lines (Figure 8C, 786-O-Res and Caki-1-Res). The inhibition efficiency of MX2 in cell lines was validated using the PCR and sh#2 was selected for further experiments (Figure 8D). Colony formation assay indicated that the knockdown of MX2 could remarkably hamper the proliferation ability of 786-O-Res and Caki-1-Res when exposed to sunitinib (Figure 8E).

## Discussion

With the change in the comprehensive environment, the incidence rate of ccRCC is increasing year by year (Jonasch et al., 2021). Advanced RCC is mainly treated with drugs, and it is not sensitive to radiotherapy and has a poor effect on chemotherapy (Barata and Rini, 2017). Although non-specific immunotherapy is beneficial to some patients with advanced RCC, it has little clinical benefit in most cases and obvious toxic exposure (Barata and Rini, 2017). Sunitinib has effectively improved the survival performance of patients with RCC, with relatively small side effects, and is currently the main means of drug treatment for renal cancer (Barata and Rini, 2017). However, in practical clinical application, some patients receiving sunitinib treatment often have acquired drug resistance, which limits their therapeutic benefits (Larroquette et al., 2021).

In this study, through publicly available data and high-quality analysis, we deeply explored the potential biological mechanisms that affect the resistance of sunitinib. Detailed, data from GSE64052, GSE76068 and TCGA were extracted. We identified the IFITM1, IL6, MX2, PCOLCE2, RSAD2 and SLC2A3 were associated with sunitinib resistance. Single-cell analysis, prognosis analysis and m6A regulatory network were conducted to investigate their role. Moreover, the MX2 was selected for further analysis, including its biological role and effect on the ccRCC microenvironment. Interestingly, we noticed that MX2 might be an immune-related gene that could affect the response rate of immunotherapy. Then, *in vitro* experiments validated the overexpression of MX2 in sunitinib-resistance cells. Colony formation assay indicated that the knockdown of MX2 could remarkably inhibit the proliferation ability of 786-O-Res and Caki-1-Res when exposed to sunitinib.

Six genes were identified to induce sunitinib resistance in ccRCC, including IFITM1, IL6, MX2, PCOLCE2, RSAD2 and SLC2A3. Some of these genes have been reported to play an important role in cancer. Provance and their colleagues found that the IFITM1 could be affected by crosstalk between the NF-κB and interferon-alpha and regulated breast cancer progression (Provance et al., 2021). Lee and their colleagues noticed that the IFITM1 affected gastric cancer pathological characteristics through epigenetic regulation (Lee et al., 2012). Yu and their colleagues indicated that the IFITM1 could facilitate colon cancer metastasis by regulating CAV-1 (Yu et al., 2015). Yao and their colleagues found that the SLC2A3 could facilitate M2 macrophage infiltration by inducing glycolysis reprogramming (Yao et al., 2020). Liu and their colleagues demonstrated that the SLC2A3 could lead to the reduction of vitamin C uptake, therefore inhibiting leukemia development (Liu et al., 2020). Juraleviciute and their colleagues noticed that the MX2 could regulate the XAF1 and make the melanoma cells sensitive to targeted therapy (Juraleviciute et al., 2021). Wang and their colleagues found that the MX2 could suppress the glioblastoma progression through ERK/P38/NF-κB signaling (Wang et al., 2019b). Our results provide a reference for revealing the mode of action of these genes in ccRCC. Meanwhile, we deeply and comprehensively analyzed the role pattern of MX2 in ccRCC, and validated its influence on sunitinib resistance through *in vitro* experiments, making it a potential clinical target.

We found that these sunitinib-resistant related genes were regulated by multiple m6A regulators. The m6A epigenetic modification has also been reported to be related to sunitinib resistance. Chen and their colleagues noticed that TRAF1 can contribute to sunitinib resistance based on the METTL14 and m6A modifications (Chen et al., 2022). Li and their colleagues noticed that the level of YTHDC1 was downregulated by YY1/HDAC2 and could regulate the sunitinib resistance targeting the ANXA1-MAPK pathway (Li et al., 2022).

Correlation analysis showed that MX2 was positively correlated with endothelial cell_EPIC, macrophages M2_QUANTISEQ, monocyte_XCELL, Tregs_QUANTISEQ, yet negatively correlated with the NK cell_QUANTISEQ and B cell plasma_XCELL. Previous studies have reported the relationship between these cells and the progression of ccRCC. For example, van Hooren and their colleagues noticed that agonistic CD40-antibody could be enhanced by sunitinib through reducing MDSCs, increasing endothelial activation, and enhancing T cell recruitment (van Hooren et al., 2016). Dannenmann and their colleagues found that the tumor-associated macrophages could destroy the function of T cells and reduce the survival rate of ccRCC (Dannenmann et al., 2013). Xu and their colleagues found that HK3 could facilitate the immune escape of ccRCC by inducing monocyte infiltration (Xu et al., 2021). Our results indicate that MX2 may complete the remodeling of the tumor microenvironment by affecting the infiltration level of these cells and then play its biological role.

Although our article provides a biological explanation for sunitinib resistance, some limitations still need to be noted. Firstly, the result from GSE64052 and GSE76068 was only at the cell level. However, due to the complex regulatory mechanism *in vivo*, our conclusions should be subsequently validated *in vivo* models. Secondly, the deep biological mechanism of MX2 in ccRCC still needs to be explored.

## Conclusion

In summary, through publicly available data and high-quality analysis, we deeply explored the potential biological mechanisms that affect the resistance of sunitinib. MX2 was selected for further analysis, including its biological role and effect on the ccRCC microenvironment. Finally, *in vitro* experiments were used to validate its role in ccRCC.

## Data Availability

The original contributions presented in the study are included in the article/Supplementary Materials, further inquiries can be directed to the corresponding authors.
