# Supplementary material for: The m6A-regulation and single cell effect pattern in sunitinib resistance on clear cell renal cell carcinoma: Identification and validation of targets
Source: Front Pharmacol. 2023 Mar 31;14:1131610. doi: 10.3389/fphar.2023.1131610 (PMC10102343; doi:10.3389/fphar.2023.1131610)
Supplement: Supplementary file 1 [file Table1.docx]

| **Table S1. The baseline information of TCGA patients** | | | |
| --- | --- | --- | --- |
| Clinical terms |  | Numbers | Percentage (%) |
| Age | <=65 | 352 | 65.5 |
|  | >65 | 185 | 34.5 |
| Gender | Male | 346 | 64.4 |
|  | Female | 191 | 35.6 |
| Grade | G1 | 14 | 2.6 |
|  | G2 | 230 | 42.8 |
|  | G3 | 207 | 38.5 |
|  | G4 | 78 | 14.5 |
|  | Unknown | 8 | 1.5 |
| Stage | Stage I | 269 | 50.1 |
|  | Stage II | 57 | 10.6 |
|  | Stage III | 125 | 23.3 |
|  | Stage IV | 83 | 15.5 |
|  | Unknown | 3 | 0.6 |
| T-stage | T1 | 275 | 51.2 |
|  | T2 | 69 | 12.8 |
|  | T3 | 182 | 33.9 |
|  | T4 | 11 | 2.1 |
| M-stage | M0 | 426 | 79.3 |
|  | M1 | 79 | 14.7 |
|  | Unknown | 32 | 5.9 |
| N-stage | N0 | 240 | 44..7 |
|  | N1 | 17 | 3.2 |
|  | Unknown | 280 | 52.1 |
