# Supplementary figures and images for: The m6A-regulation and single cell effect pattern in sunitinib resistance on clear cell renal cell carcinoma: Identification and validation of targets
Source: Front Pharmacol. 2023 Mar 31;14:1131610. doi: 10.3389/fphar.2023.1131610 (PMC10102343; doi:10.3389/fphar.2023.1131610)

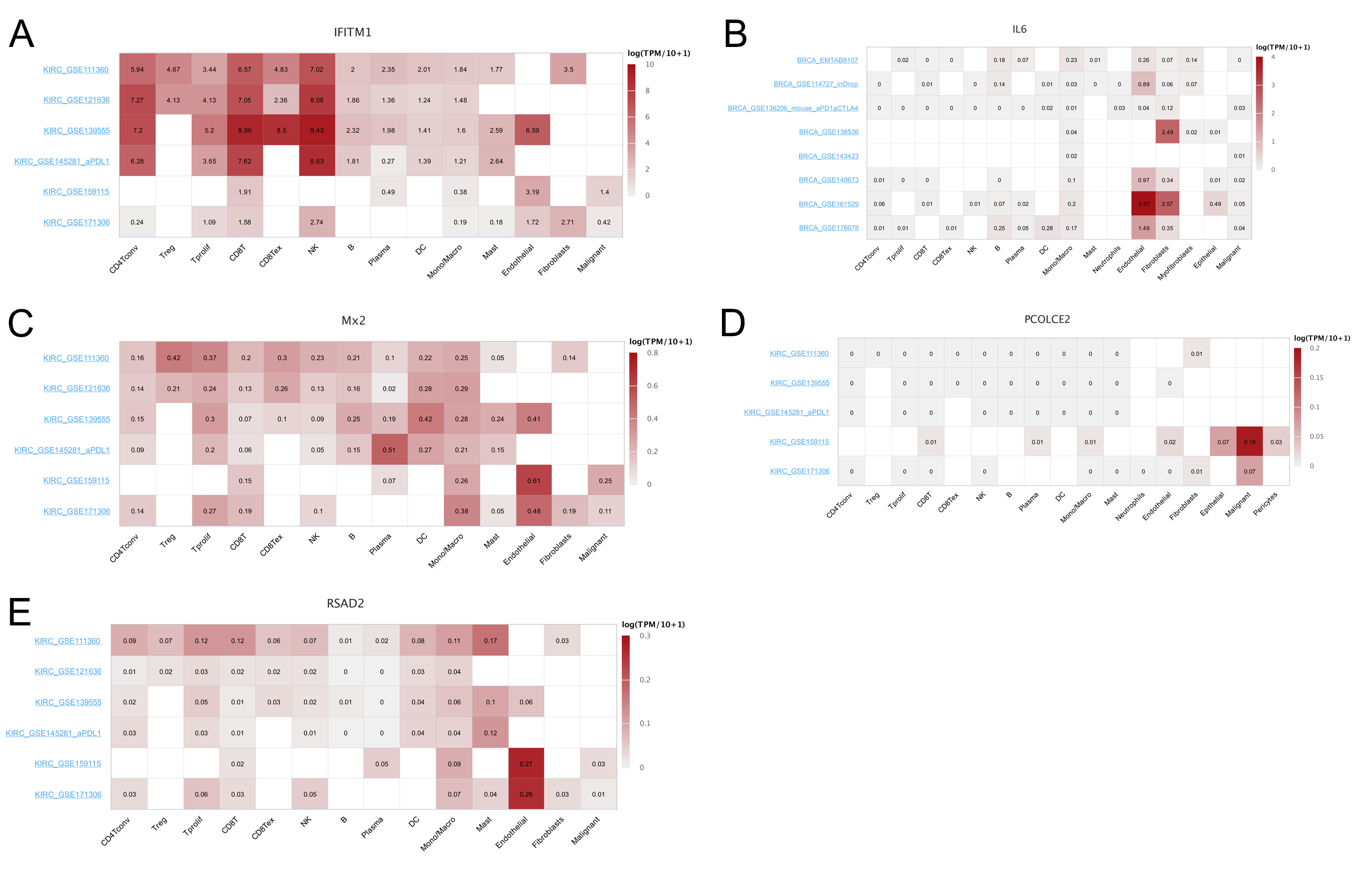

Supplement: Supplementary file 2 [file Image2.TIF]

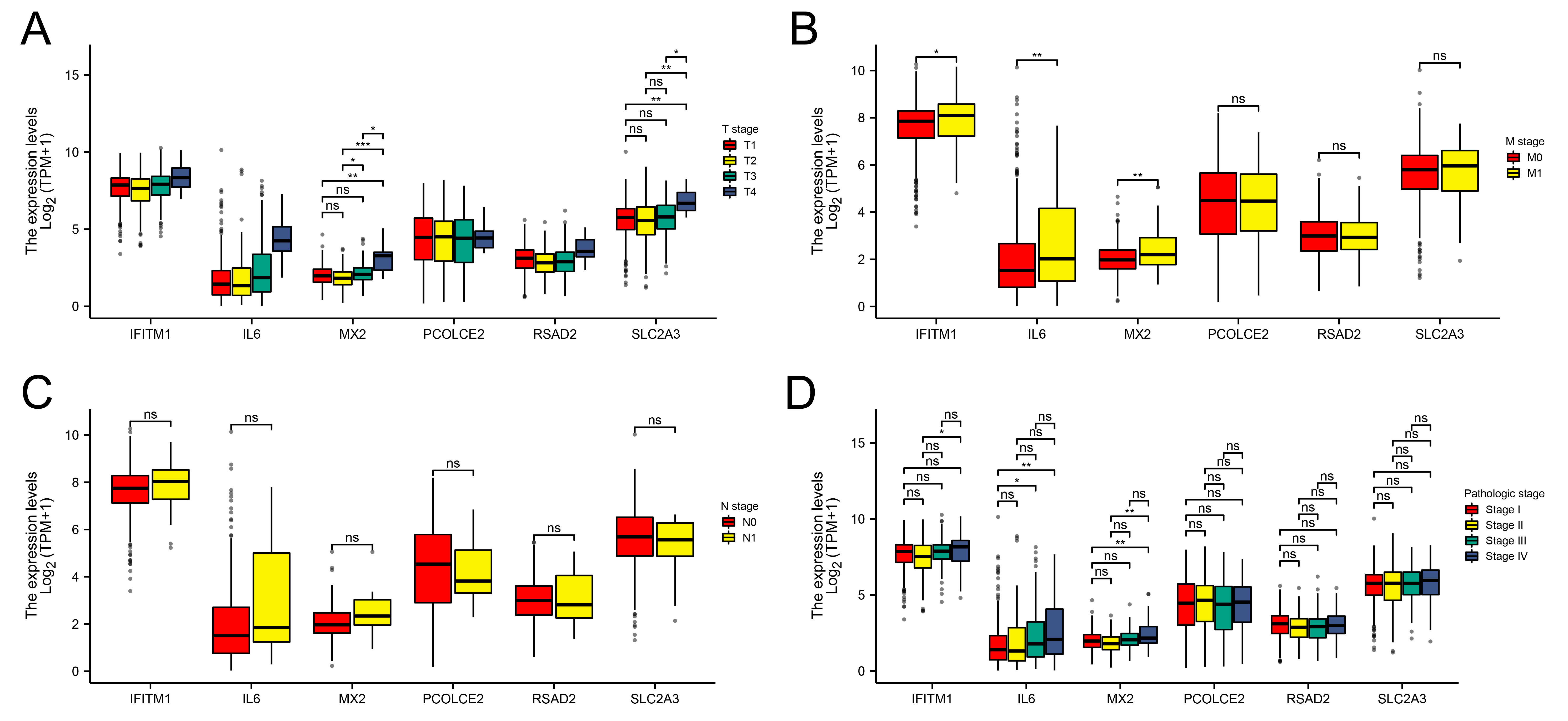

Supplement: Supplementary file 3 [file Image1.TIF]
